# Epsin15 Homology Domains: Role in the Pathogenesis of Pulmonary Arterial Hypertension

**DOI:** 10.3389/fphys.2018.01393

**Published:** 2018-10-02

**Authors:** Dan Predescu, Shanshan Qin, Monal Patel, Cristina Bardita, Rabia Bhalli, Sanda Predescu

**Affiliations:** Division of Pulmonary Medicine, Critical Care and Sleep Medicine, Department of Internal Medicine, Rush Medical College, Rush University, Chicago, IL, United States

**Keywords:** intersectin, pulmonary arterial hypertension, endocytic deficiency, alternative transport pathways, non-conventional endocytic/transcytotic structures, EH-binding protein 1, cortical actin

## Abstract

Intersectin-1s (ITSN) deficiency and expression of a biologically active ITSN fragment, result of granzyme B cleavage under inflammatory conditions associated with pulmonary arterial hypertension (PAH), are characteristics of lung tissue of human and animal models of PAH. Recently, we have shown that this ITSN fragment comprising two Epsin15 homology domains (EH_ITSN_) triggers endothelial cell (EC) proliferation and the plexiform arteriopathy in PAH. Limited evidence also indicates that the EH domains of endocytic proteins such as ITSN, upregulate compensatory endocytic pathways in cells with impaired vesicular trafficking. Thus, we sought to investigate whether the EH_ITSN_ may be involved in this compensatory mechanism for improving the EC endocytic dysfunction induced by ITSN deficiency and possibly contribute to PAH pathogenesis. We used stably-transfected human pulmonary artery ECs expressing the Myc-EH_ITSN_ (EC_EH-ITSN_) and ITSN knockout heterozygous mice (K0^*ITSN+/-*^) transduced with the Myc-EH_ITSN_, in conjunction with functional assays: the biotin assay for caveolae internalization and 8 nm gold (Au)- and dinitrophenylated (DNP)-albumin perfusion of murine lung microvasculature. Pulmonary artery ECs of PAH patients (EC_PAH_), ITSN knockdown ECs (EC_KD-ITSN_), the monocrotaline (MCT)-induced mouse and rat models of PAH, as well as untreated animals, served as controls. ELISA via streptavidin-HRP or anti-DNP antibody (Ab), applied on ECs and lung lysates indicated greater than 30% increase in biotin internalization in EC_EH-ITSN_ compared to EC_Ctrl_. Despite their endocytic deficiency, EC_PAH_ internalized biotin similar to EC_Ctrl_ which is twofold higher compared to EC_KD-ITSN_. Moreover, the lung microvascular bed of Myc-EH_ITSN_-transduced mice and MCT-treated animals showed greater than twofold increase in DNP-BSA transendothelial transport, all compared to untreated controls. Electron microscopy (EM) revealed the increased occurrence of non-conventional endocytic/transcytotic structures (i.e., caveolae clusters, tubulo-vesicular and enlarged endocytic structures, membranous rings), usually underrepresented. Most of these structures were labeled by Au-BSA, consistent with their involvement in the transendothelial transport. Furthermore, ITSN deficiency and EH_ITSN_ expression alter the subcellular localization of the EH-binding protein 1 (EHBP1) and cortical actin organization, altogether supporting the increase occurrence/trafficking of the alternative endocytic structures. Thus, the EH_ITSN_ by shifting the physiological vesicular (caveolae) transport toward the alternative endocytic pathways is a significant contributor to the dysfunctional molecular phenotype of EC_PAH_.

## Introduction

Patho-physiologically, pulmonary arterial hypertension (PAH) is defined by mean pulmonary arterial pressure exceeding the upper limits of typical values, i.e., ≥ 25 mmHg at rest, as result of extensive pulmonary vascular dysfunction (Stenmark et al., 2009; Sehgal and Lee, 2011). As a multiple etiology disease, PAH is triggered by diverse internal and/or external factors and it is perpetuated by a plethora of signaling events and altered cellular interactions at the level of lung vascular bed (Tuder et al., 2001, 2007, 2009). At the cellular level, PAH affects all segments of the pulmonary vascular bed (Chazova et al., 1995). Concentric and eccentric intimal thickening, medial hypertrophy, lumen obliteration and recanalization affect the pulmonary arteries. Modest venous hypertrophy due to increase in the intimal and adventitial thickness and capillary bed rarefaction are among the structural changes of the pulmonary vasculature in PAH, as well (Chazova et al., 1995). In most typical cases, an endothelial driven patchy proliferation of intima and formation of vascular lesions occur (Chazova et al., 1995). The plexiform lesions, hallmark of established PAH, are found at branching points in the small pulmonary arterioles; they are glomeruloid-like vascular structures, lumen-obliterating and composed predominantly of actively dividing and apoptosis-resistant endothelial cell (ECs) (Tuder et al., 1994; Sakao et al., 2005).

The plexiform arteriopathy, the rarefaction of lung capillaries and the venous hypertrophy, as leading drivers of PAH, depend on the status of pulmonary endothelium. The ECs that form the endothelial layer to separate the blood vessel lumen from the rest of the body were considered inert bystanders for an extended period (Tuder et al., 1994). However, the slow dividing and metabolically stable ECs, have the genomic fitness to switch to a migratory, proliferative, apoptotic-resistant and metabolically active phenotype in response to extra- and intracellular signals (Stevens and Gillespie, 2007). Thus, any dysfunction of ECs, permanently exposed to significant variations in physiological parameters (tension, stretch, shear) and to different chemical composition of the surroundings (blood on the luminal and the interstitial fluid on the abluminal side) is at the core of most vascular pathologies, PAH included. Current evidence indicates that paradoxical increase in EC apoptosis followed by exuberant proliferation, and formation of obliterative cellular lesions contribute to the severe vasculopathy in PAH (Long et al., 2015; Zhou et al., 2016). Additional factors such as defects in caveolae trafficking and cytoskeletal organization as well as increased vascular permeability have been linked to PAH pathology (Johnson et al., 2012; Prewitt et al., 2015; Zhou et al., 2016).

Historically, the EC was among the first cell type identified endowed with an abundant population of plasmalemmal vesicles (Palade and Bruns, 1968), re-named caveolae (Rothberg et al., 1992) – involved in endocytosis, one of the fundamental mechanisms by which the cells interact and respond to environmental clues. Moreover, caveolae function in endothelial transcytosis, a process that constitutively couples endo- and exocytosis on the opposite sides of the plasma membrane (Predescu et al., 1997). Endothelial transcytosis provides the morpho-functional basis for sustaining the magnitude, speed, and adaptability of the transendothelial transport as genetically established and functionally shaped to satisfy the general necessities and local requests (Predescu et al., 2007b).

Limited evidence indicates that the EH domains of endocytic proteins confine caveolae to the plasma membrane and upregulate compensatory endocytic pathways in cells with impaired vesicular trafficking such as pulmonary EC_PAH_ (Yap et al., 2010; Stoeber et al., 2012). The EH domain which binds to peptides containing the sequence Asp-Pro-Phe (NPF) is implicated in the control of plasma membrane receptor endocytosis and the regulation of intracellular trafficking routes, with significant ramifications into transcriptional regulation and actin cytoskeletal remodeling (Polo et al., 2003).

Intersectin-1s (ITSN), a general scaffold and regulator of the endocytic machinery (Predescu et al., 2003; Knezevic et al., 2011; Patel et al., 2013) comprises two NH_2_-terminal EH domains involved in interactions with several endocytic proteins – Eps15, Stonin2, and SCAMP1 (Yamabhai et al., 1998; Fernandez-Chacon et al., 2000; Martina et al., 2001). Moreover, within the EH2 domain of ITSN there is a well-conserved granzyme B cleavage site, IDQD^271^GK (Loeb et al., 2006; Patel et al., 2013). Under inflammatory conditions associated with PAH, granzyme B cleaves ITSN and generates the EH_ITSN_, a biologically active protein fragment, present in EC_PAH_ and human lung tissue with plexiform lesions; its expression triggers EC proliferation and selection of a proliferative/plexiform EC phenotype leading to formation of complex pulmonary vasculopathy in a murine model (Patel et al., 2013, 2017). These observations suggest that the EH_ITSN_ may facilitate the excessive growth of ECs in PAH lungs not only due to its endothelial proliferative potential but also due to upregulation of alternative transport pathways to compensate for caveolae dysfunction induced by ITSN deficiency (Predescu et al., 2012). Considerations of impaired endocytosis in ECs and its consequences on the pathobiology of PAH are mostly absent; the effects of EH_ITSN_ on vesicular trafficking and its role in the regulation of endothelial permeability are not known.

The concept that endocytosis is necessary for some forms of cell signaling (Vieira et al., 1996) has evolved into the hypothesis of endocytosis as a cellular process integrated with, and essential for the execution of various cellular programs like motility, cell fate determination, cellular reprogramming and biogenesis of miRNA, cell division, and transcription (Vieira et al., 1996; Polo and Di Fiore, 2006; Sigismund et al., 2012). Caveolae are multifunctional integrators of endocytosis and signaling (Sigismund et al., 2012). In pathological conditions, dysfunctional ECs also show altered intracellular trafficking and signaling of cell surface receptors such as the transforming growth factor beta receptor 1 (TGFβR1) implicated in the pathogenesis of PAH (Garcia-Rivas et al., 2017; Graf et al., 2018). Endocytic dysfunction and non-productive assembly of the endocytic machinery alter canonical signaling pathways with detrimental consequences on EC function (Mukherjee et al., 2006; Sorkin and von Zastrow, 2009; Bardita et al., 2015).

So far, no systematic studies have assessed the extent of endothelial endocytic dysfunction, and even fewer *in vivo* analyses measured the degree to which the transendothelial transport is affected. Thus, in this study we used stably-transfected human pulmonary artery ECs expressing EC_EH-ITSN_ and EH_ITSN_-transduced K0*^ITSN^*^+^*^/-^* mice to characterize the ECs endocytic dysfunction and to understand how ITSN deficiency and the EH_ITSN_ expression participate in the development and maintenance of the dysfunctional EC_PAH_ phenotype.

## Materials and Methods

### Materials

The reagents were obtained as follows: cholesterol, dimethyl dioctadecyl ammonium bromide, bovine serum albumin (BSA), H_2_O_2_, glycerol, benzamidine, phenylmethylsulphonyl fluoride (PMSF), sodium pyrophosphate, BSA, desipramine hydrochloride, protease inhibitors cocktail, poly-lysine solution (tissue culture grade), and Hank’s solution from Sigma-Aldrich (St. Louis, MO, United States); Micro BCA protein assay kit and enhanced chemiluminescent substrate from Pierce (Rockford, IL, United States). Triton X-100, dodecylsulphate sodium salt (SDS), Tween-20, nitrocellulose membranes, and all reagents for electrophoresis from ThermoFisher Scientific (Rockford, IL, United States), ProLong Antifade reagent (Molecular Probes, Eugene, OR, United States), G418 from Mirus BIO LLC (Madison, WI, United States). The polycarbonate 50 nm filters and the mini-extruder were from Avestin, Inc., (ON, Canada). All siRNA reagents (the SMART pool siRNA, si*CONTROL*^TM^ functional non-targeting siRNA_,_ Dharmafect1 Transfection Reagent) were from ThermoFisher Scientific (Rockford, IL, United States) Paraformaldehyde (PFA), glutaraldehyde (GA), Epon 812 embedding kit and all Electron microscopy (EM) grade reagents were purchased from EM Science (Fort Washington, PA, United States). Monocrotaline (MCT) was purchased from Cayman Chemical (Ann Arbor, Michigan, MI, United States).

The following primary antibodies (Ab) were used: rabbit anti-actin monoclonal Ab and ITSN polyclonal Abs from Sigma-Aldrich (St. Louis, MO, United States), ITSN mAb from BD Transduction Laboratories (Lexington, KY, United States), rabbit anti-DNP polyclonal Ab and fluorophore-conjugated reporters and Alexa Fluor 350 Phalloidin from Invitrogen (Carlsbad, CA, United States), EHBP1 monoclonal Ab from Santa Cruz (Santa Cruz, CA, United States) and anti-Myc monoclonal Ab from Cell Signaling (Danvers, MA, United States). SureBlue Reserve TMB Microwell Peroxidase Substrate was from KPL (Gaithersburg, MA, United States). Affinity purified F(ab’)-2 goat anti-mouse and donkey anti-rabbit IgGs, reporter Abs were from Bethyl Laboratories Inc. (Montgomery, TX, United States) and the rabbit TrueBlot anti-rabbit IgG horseradish peroxidase (HRP) was from Rockland Immunochemicals (Limerick, PA, United States).

### Animals

All animal (rodent) studies were performed according to the guidelines of Rush University Institutional Animal Care and Use Committee. K0*^ITSN^*^+/-^ mice, 129SV/J genetic background, were kindly provided by Dr. Melanie Pritchard (Monash University, Clayton, Australia). Breeding colonies were maintained in the Rush University animal facility. All mice, 6 -to 8-weeks-old, 20 g – 30 g, were kept under standardized housing and feeding conditions. All experiments were done under anesthesia using ketamine (60 mg/kg), acepromazine (2.5 mg/kg) + xylazine (2.5 mg/kg), in 0.1 – 0.2 mL sterile PBS. Mice were genotyped by tail snipping standard procedure (Patel et al., 2017). All experiments were repeated at least three times. No mouse mortality occurred during the study. MCT-mice were generated as previously described (Patel et al., 2013). Wild-type and MCT-treated rats were kindly provided by Dr. Jiwang Chen, University of Illinois at Chicago. All efforts were made to minimize suffering; in addition all studies using rodents adhered to APS’s Guiding Principles in the Care and Use of Vertebrate Animals in Research and Training and were performed according with the Rush University IACUC approved protocol number 14-023.

### Cationic Liposomes

Cationic liposomes were prepared using dimethyl dioctadecyl ammonium bromide and cholesterol by rotary evaporation of lipid solutions in chloroform, at 42°C, under vacuum (Miyawaki-Shimizu et al., 2006; Predescu et al., 2012). Briefly, the lipid film was rehydrated for 1 h at RT in 5% dextrose followed by sonication and sterile filtration through 0.45 μm and 0.22 μm membranes. Then, the suspension was extruded through the 50 nm polycarbonate filter mounted on the mini-extruder device, to assure unilamellar vesicles formation. DNA-liposomes complexes were prepared using 8 nmoles liposomes: 1 μg Myc-EH_ITSN_ DNA (amino acids 1 – 271) cloned into the pReceiver/Myc-M43 vector (GeneCopoeia, Rockville, MD, United States), a ratio found in previous studies to induce efficient protein expression without pulmonary toxicity (Patel et al., 2017). Long-term Myc-EH_ITSN_ protein expression in mouse lungs was achieved by repeated Myc-EH_ITSN_ DNA-liposome delivery via retro-orbital injections, every 48 h, for 18 days. A mutant EH_ITSN-W263A_ fragment in which the tryptophan (W) 263 was substituted with alanine (A) was cloned into the same vector and used as control (Patel et al., 2017). At the end of the treatment period, mice were anesthetized by i.p. delivery of anesthetic mixture; lung tissue was harvested and processed for histochemistry, morphological and biochemical analyses.

### Preparation of Lung Tissue Lysates

For protein extraction, the mouse lung tissue was homogenized in a buffer containing 20 mM Tris–HCl, pH 7.4, 150 mM NaCl, 1 mM PMSF and protease inhibitors, using a Brinkman Polytron homogenizer (Brinkman, Oxford, CT, United States). Total lysates were prepared by adding SDS and Triton X-100 to a final concentration of 0.3 and 1%, respectively, with gently mixing for 2 h at 4°C. The resulting lysates were clarified by centrifugation (191,500 ×*g*, 45 min, 4°C) in a Beckman Optima Max-XP ultracentrifuge with a TLA-55 rotor (Beckman, Indianapolis, IN, United States). Protein concentration was determined by the micro BCA method with BSA as standard.

### Tracer Preparation and Perfusion

Colloidal gold particles with an average diameter of 8 nm, were prepared (Predescu et al., 1998). Gold-tagged BSA (Au-BSA) was obtained by stabilizing colloidal gold suspensions with BSA (800 μg/ml) followed by 200 μg/ml poly l-glutamic acid, as secondary stabilization. The stabilized solutions were kept as stock and diluted just before use in PBS to *A*_520_ = 1. BSA was also tagged with DNP (DNP-BSA) and perfused to mouse and rat lung microvasculature to assess the permeability to albumin (Predescu et al., 1994, 1997, 1998). Quantitative assessment of DNP-BSA transendothelial transport was done by ELISA via anti-DNP Ab using a standard curve generated with known amounts of the same tracer (Predescu et al., 1997)

### EC Culture and Transfection

Pulmonary artery ECs (Lonza, Walkersville, Inc., MD, United States), and EC_PAH_ (kindly provided by Drs. Serpil Erzurum and Suzy Comhair, Lerner Research Institute, Cleveland Clinic, Cleveland OH, United States) passages 3 – 5, were grown in EBM-2 and medium 199-supplemented with 20% fetal bovine serum as previously described (Predescu et al., 2003; Patel et al., 2013). The EC_PAH_ were harvested and cultured as described in Comhair et al. (2012) from 3 PAH patients; CCF-005: Female, White, non-Hispanic, 47-year-old, idiopathic PAH; CC-027: Female, White, non-Hispanic 22-year-old, chronic thromboembolic PAH; CC-016: Male, White, non-Hispanic 27-year-old, idiopathic PAH). For stable transfection, Myc-EH_ITSN_ transiently transfected ECs were grown in 100 cm^2^ dishes with 1 mg/ml G418. HAM’s F12 medium containing 1 mg/ml G418 was replenished every other day for about 2 weeks until stable transfected ECs (EC_EH-ITSN_) began to grow. At that point the concentration of G418 in HAM’s F12 medium was switched to 800 μg/ml for a week and then maintained at 500 μg/ml. ECs transfected with the empty Myc-M43 vector (EC_Myc-M43_) and with the mutant EH_W263A_ (EC_EH-W263A_) were used as controls.

For ITSN knockdown, the individual siGENOME duplex most efficient in knocking down ITSN protein expression (siRNA_ITSN_), GGACAUAGUUGUACUGAAAUU (sense sequence) and 5′ –UUUCAGUACAACUAUGUCCUU (antisense), was selected from the SMART pool siRNA reagents and delivered in cultured ECs (Predescu et al., 2007a; Bardita et al., 2015), using Dharmafect1 transfection reagent according to the manufacturer’s instructions. The si*CONTROL*^TM^ functional non-targeting siRNA sequence 5′- UAGCGACUAA ACACAUCAA-3′ was used as control for potential secondary effects caused by siRNA transfection. The siGloCyclophilin B siRNA was used as a control for efficient transfection and to evaluate any off-target effects caused by the introduction of siRNA_ITSN_ in ECs.

### Western Blot (WB), Immunoprecipitation and Densitometry

Endothelial cells grown on Petri dishes were collected and solubilized in lysis buffer (20 mM Tris–HCl, pH 7.4, 150 mM NaCl, 0.3% SDS, 1% (w/v) Triton X-100, 1 mM Na_3_VO_4_, 1 mM PMSF, and protease inhibitors) for 1 h at RT, under gentle agitation. The resulting lysate was clarified by centrifugation in a Beckman Optima Max-XP ultracentrifuge with a TLA-55 rotor at 45,000 rpm for 45 min at 4°C. Protein concentration was determined using the micro-BCA method with a BSA standard. Protein samples normalized for total protein were subjected to SDS–PAGE and transferred to nitrocellulose membranes. Strips of nitrocellulose membranes were incubated with the primary and reporter Abs and processed as described (Predescu et al., 2007a). The reaction was visualized using an enhanced chemiluminescent substrate and HyBlot CL films. Representative films were subjected to densitometry using NIH ImageJ1.37v software.

For immunoprecipitation studies, 250 μg total protein from ECs lysates were precleared using a rabbit IgG followed by protein A/G agarose beads, for 1 h each, at RT. The resulting supernatant was incubated overnight at 4°C with anti-EHBP1 or ITSN Abs, followed by incubation 1 h, at RT with protein A/G. Beads were washed extensively, and after solubilization in electrophoresis sample buffer, the immunoprecipitates were resolved on 5 – 20% SDS–PAGE, electrotransfered to nitrocellulose membranes and analyzed by WB.

### Biotin Assay for Caveolae Internalization

Control and transfected cells grown on plastic Petri dishes or glass coverslips were washed with ice-cold PBS and then incubated with 0.5 mg/ml cleavable biotin reagent (Predescu et al., 2003). Biotinylated cell surface proteins were internalized for 30 min, at 37°C. Biotinylated proteins still at the cell surface after 30 min were reduced with glutathione and the cells were further processed for morphological analysis or lysed for biochemical investigation. For imaging, ECs grown on glass coverslips were washed in PBS (5 × 2 min), fixed and permeabilized (methanol, 7 min, -20°C), quenched (PBS + 1% BSA; 1 h, RT), and incubated with NeutrAvidin Alexa Fluor 594 (1 h, at RT). ECs were rewashed (5 × 2 min), mounted on glass slides using ProLong Antifade reagent, examined, and photographed Zeiss AxioImager M1 motorized upright microscope equipped with AxioCam ICc1 R3 RGB color digital camera (Carl Zeiss MicroImaging, Inc., Thornwood, NY, United States) (Predescu et al., 2003). For biochemical studies, ECs lysates, expected to contain the internalized biotinylated cell surface proteins were prepared as above. The amounts of biotin labeled proteins were assessed by ELISA via streptavidin-HRP. For quantitative assessment of biotin molecules in the EC supernatants, a standard curve was generated using known concentrations of BSA-biotin. The average number of biotin molecules present in each cell lysate was determined at a series of decreasing concentrations from the linear part of the curve obtained by successively diluting a standard volume (100 μl) from each lysate and normalized per mg total protein.

### Immunostaining

Immunofluorescent staining of ECs grown on coverslips was performed as described previously (Bardita et al., 2015). ITSN1 Ab (Prestige, Sigma-Aldrich, United States), was used at 1:500 dilution and EHBP1 Ab at 1:50 dilution in 0.1% BSA in PBS, whereas Alexa Fluor 350 Phalloidin was used at 6.6 μM, as per manufacturer’s instructions. Incubation of ECs with isotype-matched IgGs or omission of the primary Ab was used as controls for Ab specificity. Each set of experiments was performed in triplicate.

### Transmission Electron Microscopy (TEM)

The EC monolayers were fixed in 2% PFA + 0.5% GA + 1% tannic acid in 0.1 PIPES, pH 7.2, for 30 min at RT; all specimens were post-fixed in 2% OsO_4_ in acetate veronal buffer, pH 6.8, for 30 min on ice (Predescu et al., 2001, 2007b). Fixed ECs were stained for 30 min with 7.5% uranyl-magnesium acetate (UA), dehydrated through increasing concentrations of ethanol (50, 70, 90, and 100%), then infiltrated with a mixture of 100% alcohol: Epon 812 (1:1) for 1 h and embedded in Epon 812. Embedded specimens were cured for 72 h at 60°C. Blocks of ∼ 0.5/0.5 cm cut off from polymerized resin were used to obtain sections ∼ 60 nm, which were stained with 7.5% UA for 5 min and saturated lead citrate for 3 min, examined and photographed in a Jeol-1220 EM.

The lung tissue was fixed *in situ* by perfusion of a fixative mixture (3% PFA, 2.5% GA in 0.1 PIPES, pH 7.2) for 15 min, and selected specimens were further fixed with the same mixture for 1 h at RT. All specimens were post-fixed in 2% OsO_4_ in acetate veronal buffer, for 1 h on ice, stained for 1 h with 7.5% UA, dehydrated through increasing concentrations of ethanol and propylene oxide as above, and embedded in Epon 812. Embedded specimens were cured for 72 h at 60°C, sections of ∼ 60 nm were stained with 7.5% UA for 5 min and saturated lead citrate for 3 min and then examined as above.

### Image and Morphometric Analyses

Single image analysis after immunostaining was performed (Predescu et al., 2003), using a new interactive web program written in the statistical programming environment R – the Squassh3C and Squassh analyst^1^. To analyze the surface density of the fluorescent puncta, the workflow functions of the program, with image data files in any format supported by Image J which is part of the open-source Fiji software^2^ installed on a Windows 10 platform of a PC, was used. Briefly, digital fluorescence images were acquired (Keren et al., 2001; Lachmanovich et al., 2003), using the AxioCam ICc1 R3 RGB color digital camera coupled to a Zeiss microscope with a Plan Apochromat 100/1.4 oil-immersion objective. Images were acquired at a 1 × 1 binning (1280 × 1020 pixels, pixel size 0.1 × 0.1 μm). For each condition a number of 50 regions of interest, randomly selected from 6 to 10 coverslips were analyzed with the particle counting and analysis feature of the mentioned software.

For EM, 6 – 8 Epon blocks were employed for thin sectioning, and six grids per block, 15 – 25 sections/grid were examined. Sections (60 – 70 nm) were obtained at random, and only cells with a full profile on the grid mesh were photographed. The images were acquired with a Gatan charge-coupled device camera. A total of 124 images for every condition, at the final magnification of ×28,000 were stacked as a queue and grid no. 3 from the Stereology Toolbox program (Morphometrix) was used to quantify the main endothelial features.

### Statistical Analysis

Data were analyzed and the comparison between groups, when needed, was done using one-way analysis of variance and Student’s *t*-test with a Bonferroni correction for multiple comparisons. All data are expressed as a mean ± standard deviation (SD). For BSA permeability, data were analyzed by one-way ANOVA followed by a *post hoc* Dunnett’s test. *p*-values < 0.05 were considered to have statistical significance.

## Results

### Expression of Myc-EH_ITSN_ Interferes With the ECs Endocytic Activity

**FIGURE 1:**
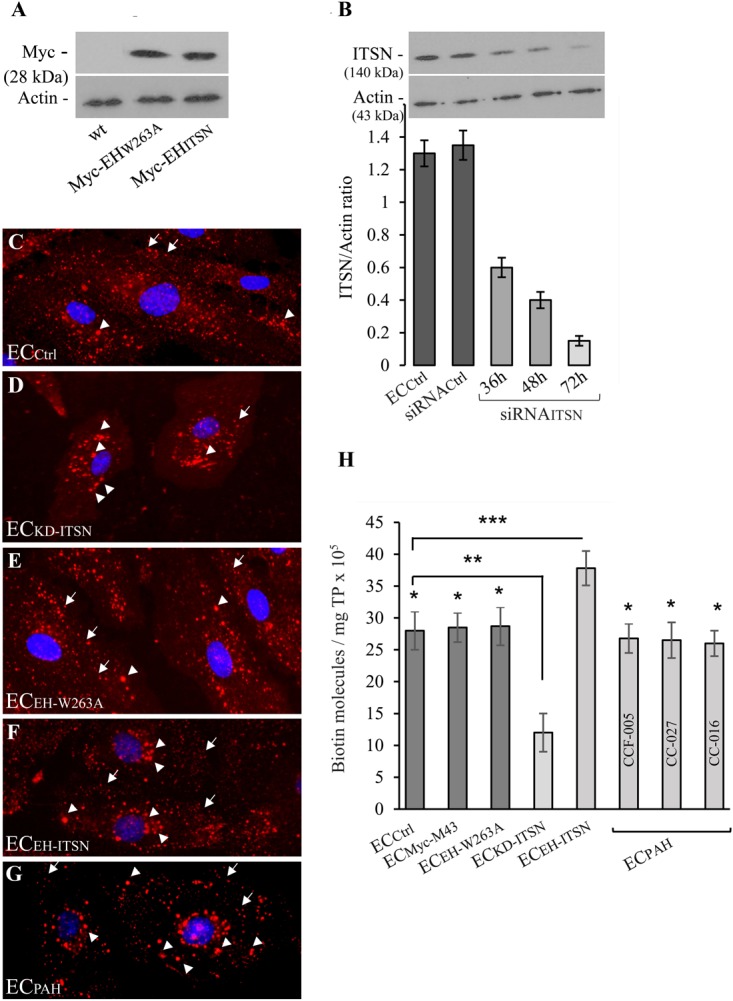
Expression of Myc-EH_ITSN_ interferes with the ECs endocytic activity. **(A)** Lysates (70 μg/lane) of EC_Ctrl_, EC_EH-W263A_, and EC_EH-ITSN_ were subjected to SDS–PAGE and WB analyses using anti-Myc Ab. **(B)** Expression of ITSN in EC_Ctrl_ (lane a) and ECs transfected with the siRNA_Ctrl_ (lane b) and EC_KD-ITSN_ (lanes c, d, e). Three time points post-siRNA_ITSN_ transfection (36, 48, and 72 h) are shown, *n* = 3; ^∗^*p* < 0.01. EC_Ctrl_ **(C)**, EC_KD-ITSN_ **(D)**, EC_EH-W263A_ **(E)**, EC_EH-ITSN_ **(F)**, and EC_PAH_ **(G)**, CCF-005 EC_PAH_ shown) were subjected to biotin internalization assay followed by NeutrAvidin Alexa Fluor 594 staining. The arrows in panels **C**–**G** indicate the fine biotin-NeutrAvidin Alexa Fluor 594 punctate pattern, suggestive of biotin association with endothelial vesicular carriers. The arrowheads in **D**–**G** point toward large fluorescent puncta assumed to be the morphological intermediates of the alternative transport pathways. **(H)** The number of biotin molecules in the final supernates of ECs lysates, as indicated. Biotinylated cell surface proteins were detected by ELISA via streptavidin-HRP. Three different EC_PAH_ preparations (CCF-005, CC-027, CC-016, as indicated) were used. Results are the averages ± SD of three different experiments performed in triplicates. ^∗^*p* < 0.001; ^∗∗^*p* < 0.01; ^∗∗∗^*p* < 0.05 vs. EC_*Ctrl.*_

The observations that the EC_PAH_ and human PAH lung tissue with plexiform lesions show reduced levels of ITSN and expression of the EH_ITSN_ protein fragment (Patel et al., 2013), led as to evaluate the endocytic activity of EC_PAH_ as well as of stable transfected EC_EH-ITSN_. Non-transfected ECs (EC_Ctrl_), ITSN-deficient ECs (EC_KD-ITSN_) and ECs transfected with the empty Myc-M43 vector (EC_Myc-M43_) were used for comparison. The specificity of EH_ITSN_ effects was addressed by transfection of ECs with the mutant EH_W263A_ [the substitution W263A reduces protein interactions of the EH domains with the NPF motif beyond detection (de Beer et al., 1998)]. Efficient expression of Myc-EH_W263A_ and Myc-EH_ITSN_, 48 h post-transfection is documented in **Figure 1A**. No immunoreactivity for Myc tag is detected in the EC_Ctrl_ lysates, **Figure 1A**. As ITSN knockdown, 72 h post-siRNA_ITSN_ transfection triggers EC apoptotic death, the EC_KD-ITSN_ were used 38 – 40h post-siRNA_ITSN_ delivery, when ITSN protein expression is 50% lower compared to EC_Ctrl_ (**Figure 1B**), and ECs are not yet apoptotic (Predescu et al., 2007a; Bardita et al., 2015). Controls and EC_EH-ITSN_ were subjected to the biotin assay for caveolae internalization followed by detection of the internalized biotinylated proteins by fluorescent imaging via neutrAvidin Alexa Fluor 594. All experimental conditions used, EC_Ctrl_ (**Figure 1C**), EC_KD-ITSN_ (**Figure 1D**), EC_EH-W263A_ (**Figure 1E**), EC_EH-ITSN_ (**Figure 1F**), EC_PAH_ (**Figure 1G**), and EC_Myc-M43_ (not shown) revealed fine puncta at the cell surface and scattered all over the cytoplasm (**Figures 1C–G**, arrows). Two significant differences were noted in the pattern of biotin/neutrAvidin Alexa Fluor 594 labeling among the experimental conditions used: (i) the fine punctate labeling, indicative of biotin association with endothelial vesicular carriers, was less pronounced in EC_KD-ITSN_ and EC_PAH_ due to the reduced caveolae number (consequence of ITSN deficiency), and ii) the frequency of the large fluorescent puncta showed an increase in both EC_EH-ITSN_ and EC_PAH_, compared to EC_Ctrl_. This increase in large puncta is consistent with the ability of the EH domains to upregulate morphological intermediates (large endocytic structures, membranous rings, and vesiculo-tubular elements) of the compensatory endocytic pathways (**Figures 1F,G**, arrowheads). A slight increase in large fluorescent puncta is also detected in the EC_KD-ITSN_ (**Figure 1D**, arrowheads). No detectable differences were noted between the biotin/neutrAvidin staining pattern between EC_Ctrl_ and EC_EH-W263A_ (**Figures 1C,E**). Under control conditions, the large endocytic structures are underrepresented and thereby the large fluorescent puncta are only seldom detected (**Figures 1C,E**, arrowheads).

Quantification of internalized biotin by ELISA via streptavidin-HRP indicated a 26% increase in biotin uptake in the EC_EH-ITSN_ compared to EC_Ctrl_ (**Figure 1H**; *n* = 8; *p* < 0.001); The lysates of three different EC_PAH_ lines showed on average only 6% decrease in the number of biotin molecules compared to EC_Ctrl_ (**Figure 1H**, *n* = 9; *p* < 0.05), while 50% ITSN knockdown (**Figure 1B**, inset) induced 75% inhibition of biotin uptake (**Figure 1H**; *n* = 5; *p* < 0.05). The minimal inhibition in biotin internalization in EC_PAH_, a stable phenotype characterized by ITSN deficiency and EH_ITSN_ expression, implies that despite impaired caveolae endocytosis caused by lack of ITSN, the functional upregulation of compensatory transport pathways accounts for a still highly efficient endocytic activity. This observation demonstrates a potential role of the EH_ITSN_ in supporting EC_PAH_ proliferation and overgrowth, and thus, the development of the disease.

**FIGURE 2:**
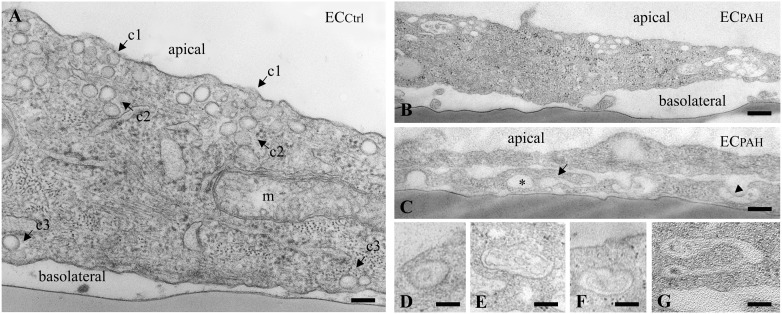
Increased occurrence of non-conventional endocytic intermediates in EC_PAH_. **(A)** Representative electron micrograph of EC_Ctrl_ illustrates a fragment of EC comprising numerous caveolae open to the apical front of the cell (c1), apparently free in the cytosol (c2) and in close proximity of the basolateral membrane (c3). A mitochondrion (m) is seen in the lower right corner. **(B,C)** Representative electron micrographs of EC_PAH_ show the reduced number of caveolae and the presence of non-conventional endocytic intermediates, i.e., an enlarged endocytic structure (**C**, asterisk), a membranous tubule (**C**, arrowhead) and a vesiculo-tubular structure (**C**, arrow). Frequently, membranous rings with diameter between 150 and 300 nm **(D–F)** and complex tubulo-vesicular structures **(G)** were detected. Bars: 100 nm **(A, D–G)**; 150 nm **(B)**; *n* = 5.

To get more insights into the effects of the EH_ITSN_ expression on the endothelial vesicular carriers and endocytic structures (morphological intermediates of compensatory endocytic pathways), we applied EM analysis of ultrathin sections prepared from randomly chosen Epon-embedded EC_PAH_. An obvious observation was the reduced number of caveolae in EC_PAH_ (**Figure 2B**) by comparison to EC_Ctrl_ (**Figure 2A**). The size of caveolae (50 – 80 nm external diameter), was not affected by the experimental manipulations performed; even the clustered caveolae preserved their dimensions. In addition, the increased presence of pleomorphic morphological structures/intermediates, deemed to function as alternative endocytic pathways was noticeable. Frequently, we detected enlarged endocytic structures (**Figure 2C**, asterisk), membranous tubules (**Figure 2C**, arrowhead), tubulo-vesicular elements (**Figures 2C** arrow,**G**) open to the apical front of the EC or with no noticeable communication with the extracellular environment and membranous rings (**Figures 2D–F**), ranging between 100 and 300 nm diameter with a narrow lumen (20 ± 4 nm). Morphometric analyses showed numerical statistical significance, **Table 1**. The increased presence of these pleomorphic morphological structures / intermediates, usually underrepresented and thought to function in alternative endocytic pathways, was confirmed in EC-EH_ITSN_ and EC_KD-ITSN_ as well, **Table 1**. Our data demonstrate ∼ 3.8-fold increase in the frequency of alternative endocytic structures in EC-EH_ITSN_ and EC_KD-ITSN_ compared to EC_Ctrl_, while EC_PAH_ show ∼ 4.5-fold increase. In addition, the number of caveolae decreases by more than 30% in all experimental conditions by comparison to EC_Ctrl_.

**Table 1:** Morphometric analyses of caveolae and alternative endocytic structures in cultured endothelial cell (ECs).

Experimental condition	Total number of caveolae	Caveolae open	Caveolae clusters	Total Rings	Rings per cell profile	Tubules/ tubulo-vesicular features	Enlarged endocytic structures (secondary lysosomes, auto-phagosomes)
EC_Ctrl_	624 ± 42	368 ± 37	6 ± 0.6	3 ± 0.5	< 0.1	1 ± 0.2	24 ± 3
EC_PAH_	387 ± 22	144 ± 31	12 ± 1.1	69 ± 9	9 ± 0.7	11 ± 0.6	52 ± 8
EC-EH_ITSN_	402 ± 34	115 ± 29	11 ± 1.9	58 ± 11	7 ± 0.5	8 ± 0.3	48 ± 7
EC_KD-ITSN_	412 ± 18	170 ± 26	14 ± 1.8	56 ± 12	7 ± 1.2	9 ± 0.8	48 ± 9

*Electron microscopy (EM) micrographs (178 for EC_Ctrl_, 237 for EC_PAH_, 198 for EC-EH_ITSN_ and 204 for EC_KD-ITSN_) with full EC profiles at a final magnification of × 30.000 were used for each experimental condition. Data were normalized per 100 μm^2^ surface and expressed as mean ± SD. n = 3 independent experiments.*

### Alternative Transport Pathways Are Upregulated and Involved in Transendothelial Transport in PAH Specimens

To address the functional effects of ITSN deficiency and EH_ITSN_ expression on ECs endocytic/transcytotic activity and on lung microvascular permeability in PAH we used our recently developed EH_ITSN_-transduced K0^*ITSN+/-*^ mouse model of plexiform arteriopathy (Patel et al., 2017). Briefly, K0^*ITSN+/-*^ mice were selected by genotyping; conventional RT-PCR applied on K0^*ITSN+/-*^ mouse lung samples, followed by densitometry, showed a 50% decrease in ITSN mRNA level by reference to wt-mice (**Supplementary Figure S1A**). Cyclophilin was used as an internal control. Similarly, ITSN protein expression was 50% lower in the K0^*ITSN+/-*^ mice compared to wt-mice, as documented by WB analyses of lung lysates, followed by densitometry. Efficient expression of the EH_ITSN_ for 21 days was achieved by repeated delivery of the Myc-EH_ITSN_ as described under Methods and illustrated in **Supplementary Figure S1B**. The PAH phenotype of these mice, right ventricular systolic pressure (RVSP) and Fulton’s index were determined as in Patel et al. (2017), and illustrated in **Supplementary Figures S1C,D**, respectively. We have also used the MCT-treated mice and rats – two widely accepted experimental animal models of PAH (Stenmark et al., 2009). As previously reported (Patel et al., 2013), the lung tissue of both MCT-mice and rats is deficient of ITSN and shows expression of the EH_ITSN_ (**Supplementary Figures S1E,F**).

**FIGURE 3:**
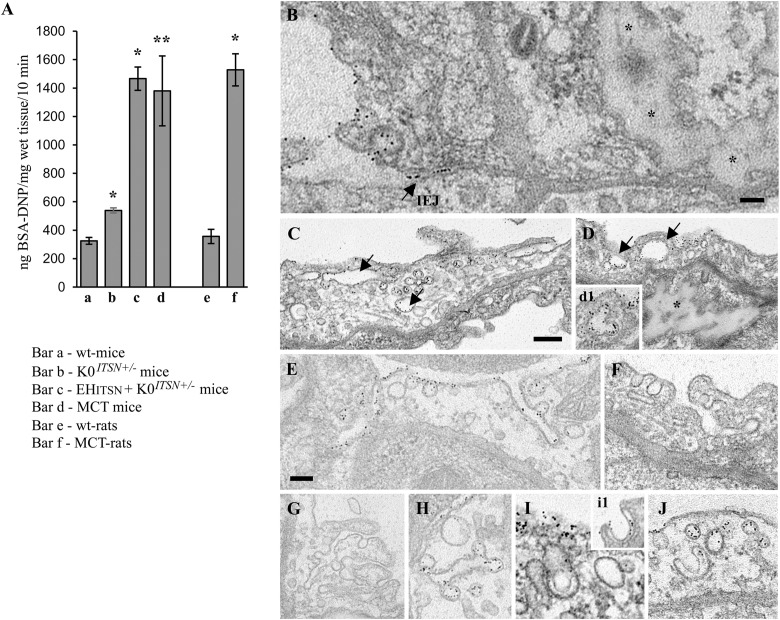
Non-conventional endocytic intermediates function in the transendothelial tracer transport. **(A)** Quantification of BSA-DNP transendothelial transport by ELISA applied on lung lysates of wt-mice **(a)**, K0^*ITSN+/-*^ mice (b), EH_ITSN_-transduced K0^*ITSN+/-*^ mice (c), and monocrotaline (MCT)-treated mice (d) as well as on lung lysates of wt-rats **(e)** and MCT-rats (f). Results are expressed in ng BSA-DNP/mg total protein/10 min. *n* = 3 experiments performed in triplicates Bars ± SD. ^∗^*p* < 0.05; ^∗∗^*p* < 0.01. **(B–J)** Representative electron micrographs of ECs profiles in the lung of EH_ITSN_-transduced K0^*ITSN+/-*^ mice show a leaky IEJ labeled in the luminal introit by 8 nm Au-BSA (**B**, arrow) and proteinaceous edema (**B,D,** asterisks). Enlarged endosomes loaded with gold particles (**C,D** arrows, inset **d1**), tubular elements, some of them branching and open to the lumen **(E–G)** or with no apparent communication with the extracellular milieu **(H,J)** and membranous rings **(I,i1)** are often detected. The tubular elements are associated with caveolae-like morphology **(H)** or display at their cytosolic termini clathrin-coated vesicles **(F)**. The Electron microscopy (EM) images shown in panels **F**, **G** were selected from a mouse lung specimen not perfused with 8 nm Au-BSA. Caveolae **(I,J)** and caveolae clusters **(C)** labeled by Au-BSA are also present. Bars: 50 nm **(B)**; 100 nm **(E,F,H–J)**, 200 nm **(C,D,G)**; *n* = 3.

First, the EH_ITSN_-transduced K0^*ITSN+/-*^ mouse lung microvasculature was perfused with 10 mg/ml DNP-BSA tracer for 10 min. Wt- and the K0^*ITSN+/-*^ mice were used as controls. Lung tissue was collected, homogenized in PBS and subjected to biochemical quantification of the amounts of DNP-BSA tracer transported to the lung interstitia. ELISA via anti-DNP Ab indicated a significant increase in DNP-BSA transport in the EH_ITSN_-transduced K0^*ITSN+/-*^ mice relative to controls (i.e., 1466 ng DNP-BSA/mg total protein/10 min vs. 325.3 ng DNP-BSA/mg total protein/10 min in wt-controls and 539 ng DNP-BSA mg total protein/10 min in K0^*ITSN+/-*^ mice), consistent with endothelial barrier dysfunction, **Figure 3A**. An increase in the DNP-BSA transport by comparison to wt-control mice (812 ng DNP-BSA/mg total protein/10 min vs. 325.3 ng DNP-BSA/mg total protein/10 min) was also recorded in the MCT-induced mouse model of PAH (**Figure 3A**). Finally, when the DNP-BSA tracer was perfused through rat lung microvasculature, the quantification of the transendothelial tracer transport indicated an increase from 356 ng DNP-BSA/mg total protein/10 min to 1528 ng DNP-BSA/mg total protein/10 min (**Figure 3A**) for controls vs. MCT-treated rats.

We further analyzed by EM the morphology of interendothelial junctions (IEJs) and if the 8 nm Au-BSA tracer can escape through open IEJs. Detailed analysis of ultrathin sections prepared from randomly chosen EH_ITSN_-transduced K0^*ITSN+/-*^ mouse lung specimens showed frequently edematous areas (**Figures 3B,D**), but no IEJs penetrated by the 8 nm Au-BSA tracer (**Figure 3B**). Given the presence of the edematous areas, this observation suggested that the IEJs are leaky but their opening is not wide enough to allow the paracellular transport of the tracer. The electron micrograph in **Figure 3B** shows indeed an IEJ whose luminal introit (arrow) is heavily labeled by 8 nm Au-BSA unable to penetrate the junction and nearby an edematous area, suggestive of junctional leakiness. This finding compellingly demonstrated that the increased permeability for BSA-DNP in the lung vasculature of EH_ITSN_-transduced K0^*ITSN+/-*^ mouse is due to the leaky IEJs, restrictive to 8 nm Au-BSA; the size of Au-BSA complexes [the overall diameter of Au-BSA complexes (gold + BSA molecules) is about 20 nm while the BSA-DNP is only 6 nm, maximum molecular diameter]. A junctional opening of ∼ 6 nm, allows water, ions and small molecules to take the paracellular pathway to the interstitia, data that are in agreement with previous reports (Simionescu et al., 2002). We did not detect gold particles associated with the abluminal exit of the junction.

Another interesting observation was the frequent detection of pleomorphic morphological structures/intermediates thought to function in alternative endocytic pathways, such as enlarged endocytic structures, many of them fused with typical caveolae and heavily labeled by gold particles (**Figures 3C,D,I**, arrows,-d1), tubules and tubulo-vesicular elements open to the lumen (**Figures 3C,E–G**) or with no noticeable communication with the extracellular environment (**Figure 3G**), membranous rings (**Figures 3I-i1,J**), many of them loaded with 8 nm Au-BSA particles or open to the lumen and loading the tracer (inset i1). The rings ranged between 150 and 300 nm diameter and displayed a narrow lumen (20± 4 nm). Morphometric analyses revealed that the EH_ITSN_ administration causes 10-fold increase in the number of alternative endocytic structures, while the number of caveolae decreases by 26% compared to wt-mice (**Table 2**).

**Table 2:** Morphometric analyses of caveolae and alternative endocytic structures in wt-, EH_ITSN_-transduced K0^*ITSN+/-*^ and MCT-treated mice.

Experimental condition	Total number of caveolae (per 100 μm^2^)	Caveolae Clusters	Membranous rings	Tubules/ tubulo-vesicular features	Enlarged endocytic structures (secondary lysosomes, auto-phagosomes)
Wt-mice	3700 ± 55	3.2 ± 0.6	1.8 ± 0.5	0.8 ± 0.3	9.3 ± 1.5
EH_ITSN_-K0^*ITSN+/-*^ mice	2738 ± 32	31 ± 4.7	21.6 ± 2.4	24.2 ± 3.2	76.3 ± 14
MCT mice	2911 ± 51	22.1 ± 6.2	10.2 ± 2.4	10.4 ± 3.8	139.5 ± 21

*Aggregated areas of 625 μm^2^ for wt-mice (164 arteriolar, 192 capillary and 107 venular profiles were examined), 817 μm^2^ for the EH_ITSN_-transduced K0^ITSN+/-^ mice (128 arteriolar, 255 capillary and 172 venular profiles were examined) and 417 μm^2^ for the MCT-treated mice (125 arteriolar, 95 full capillary and 102 venular profiles examined) were used for counting. The data are normalized per 100 μm^2^ EC surface. All arterioles with diameters between 60 and 120 μm and venules up to 70 μm were included in the sampling. There are no detectable (less than 0.1%) secondary lysosomes in arterioles or capillaries under physiological conditions, while the venular endothelium displays less than 2%. Values are means ± SD; n = 3.*

**FIGURE 4:**
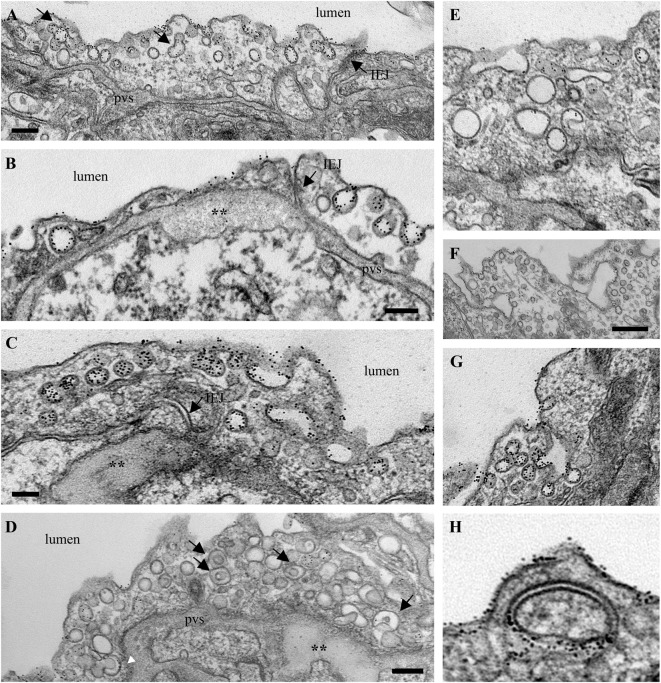
Increased occurrence of non-conventional endocytic intermediates in the MCT-treated mouse lungs. **(A)** Representative electron micrographs of a lung EC fragment of a wt-mouse. Note the caveolae labeled by the 8 nm Au-BSA tracer, a tight IEJ (arrow) and the narrow perivascular space (pvs). Leaky IEJs (**B,C**, arrows) and perivascular edema (**B,C,D,** asterisks), enlarged endocytic structures **(C,E)**, membranous rings with diameters between 100 and 400 nm (**D**, arrows, **H**), tubules open to the lumen and displaying at their cytosolic termini caveolae **(F,G)**, all labeled by 8 nm Au-BSA are frequently detected in MCT-treated mouse lungs. Bars: 100 nm **(B,H)**, 160 nm **(A,C,D,E,G)**, 350 nm **(F)**; *n* = 3.

To get insights into the contribution of the EH_ITSN_ to this lung PAH phenotype, we also analyzed the MCT-treated mice (**Figure 4**). Detailed analysis of ultrathin sections prepared from randomly chosen MCT-mouse lung specimens showed the same salient features identified in the EH_ITSN_-transduced K0^*ITSN+/-*^ mice: (i) caveolae heavily loaded with 8 nm Au-BSA (**Figures 4A–C**,**F**), (ii) IEJs restrictive to tracer particles (**Figures 4A,B**), (iii) edematous regions signifying that the EH_ITSN_ expression interferes with endothelial junctional integrity, (iv) enlarged endocytic structures (**Figures 4C,E**), (v) membranous rings (**Figures 4D,H**), and (vi) tubulo-vesicular structures (**Figures 4E–G**). Note that the upregulated alternative endocytic structures are actively involved in uptake and transport of tagged BSA molecules. Morphometric analyses of caveolae and alternative endocytic structures in the MCT mouse model of PAH demonstrate that MCT treatment causes 12-fold increase in the number of alternative endocytic structures by comparison to the wt-mouse (**Table 2**). Moreover, the number of caveolae decreases by 22% compared to the wt-mouse (**Table 2**).

**FIGURE 5:**
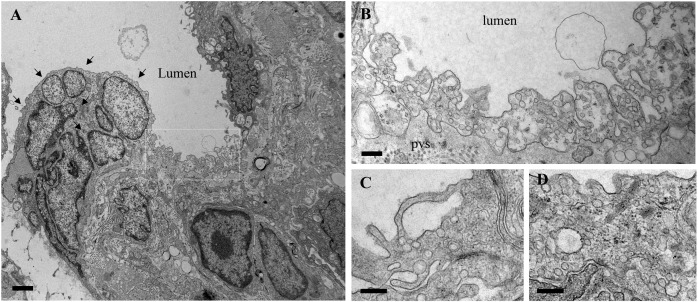
Proliferative ECs and increased occurrence of non-conventional endocytic intermediates in the MCT-treated rat lungs. **(A)** Representative electron micrographs of MCT-rat lungs perfused with 8 nm Au-BSA illustrate EC proliferation and a possible vascular lesion (arrows). **(B)** The highly magnified boxed area in A shows numerous tubular structures open to the lumen or open to the perivascular space. **(C,D)** Enlarged endocytic structures labeled by Au-BSA are often detected. Bars: 500 nm **(A)**, 250 nm **(B,C,D)**; *n* = 3.

The functional effects of ITSN deficiency and EH_ITSN_ expression on lung microvascular permeability were also assessed in the lungs of the MCT-rat model of PAH, by 10 min perfusion of 10 mg/ml DNP-BSA through lung microvasculature and quantifying by ELISA the amounts of DNP-BSA tracer transported to lung interstitial space. Biochemical quantitation of the tracer amounts transported to the lung interstitia indicated a 30% increase by comparison to untreated rats (**Figure 3**). EM analyses of ultrathin sections prepared from the lungs of the Monocrotaline (MCT)- rat model of PAH confirmed that ITSN deficiency and the presence of the EH_ITSN_ induces a similar lung phenotype as in the EH_ITSN_-transduced K0^*ITSN+/-*^ mouse (i.e., leaky IEJs and increased occurrence of pleomorphic morphological structures functioning as morphological intermediates of alternative endocytic pathways), **Figure 5**. Numerous tubulo-vesicular structures open to the lumen and the perivascular space (**Figures 5A,B**) and enlarged endocytic structures (**Figures 5C,D**), often labeled by 8 nm Au-BSA tracer were readily detected. Note, the proliferative ECs in the lumen of the vessel (**Figure 5A**, arrows), most likely part of a vascular lesion. Morphometric analyses demonstrate that MCT treatment causes 12.3-fold increase in the number of enlarged endocytic structures while caveolae number decreases by 26% by comparison to the wt-rat (**Table 3**). The substantial increase in the number of secondary lysosomes, auto-phagosomes in the MCT-treated animals mat reflect the cellular response to the MCT toxicity.

**Table 3:** Morphometric analyses of caveolae and alternative endocytic structures in wt- and MCT-treated rats.

Experimental condition	Total number of caveolae	Caveolae Clusters	Membranous rings	Tubules/ tubulo-vesicular features	Enlarged endocytic structures (secondary lysosomes, auto-phagosomes)
wt rat	4257 ± 82	5.4 ± 1.8	2.0 ± 0.8	1.1 ± 0.7	10.1 ± 2.2
MCT rat	3180 ± 88	25.2 ± 3.3	19.1 ± 4.8	18.5 ± 4.1	166.8 ± 16

*Aggregated areas of 924 μm^2^ for wt-rats (181 arterioles, 212 full capillaries and 117 venular shapes examined) and 842 μm^2^ for MCT-rats (155 arterioles, 195 capillaries and 202 venular profiles examined) were used for counting. The data are normalized per 100 μm^2^ EC surface. All arterioles with a diameter between 60 and 125 μm and venules up to 75 μm were included in the sampling for both wt- and MCT-rats. Values are means ± SD; n = 3.*

Altogether, the findings indicate that: (i) ITSN1 expression is required for maintaining the caveolae number and for their transport function in lung microvascular ECs; (ii) expression of the EH_ITSN_ interferes with endothelial junctional integrity, and (iii) expression of the EH_ITSN_ upregulates compensatory endocytic pathways, underrepresented under normal conditions.

### A Functional Partnership ITSN-EHBP1 Contributes to Actin Organization and Modulation of Endothelial Transport

**FIGURE 6:**
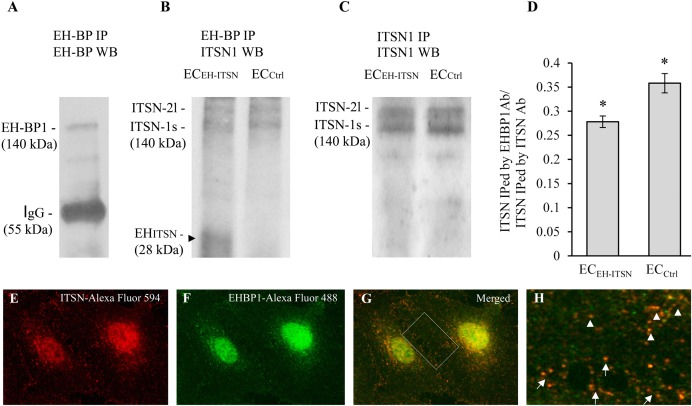
Intersectin-1s (ITSN) interacts via the EH domains with the EHBP1. ECs lysates (250 μg total protein) were subjected to immunoprecipitation with anti-EHBP1 Ab (1 μg), followed by WB with EHBP1 **(A)** and ITSN1 **(B)** Abs. EHBP1 Ab brings down the EHBP1 protein as well as ITSN. The 55 kDa immunoreactivity in panel **A** is cross-reactivity with the IgG heavy chain. The EHBP1 Ab immunoprecipitates the Myc-EH_ITSN_ from the stable transfected EC_EH-ITSN_ lysates (**B**, arrowhead). **(C)**. ECs lysates (250 μg total protein) were subjected to immunoprecipitation with anti-ITSN1 Ab (1 μg), followed by WB with ITSN1 Ab. ITSN1 Ab brings down ITSN protein in both EC_EH-ITSN_ and EC_Ctrl_ lysates. The upper ITSN immunoreactivity (190 kDa), belongs to the ITSN-2 long isoform (ITSN-2l). For immunoprecipitation studies **(B,C)**, the rabbit IgG TrueBlot Ab HRP-conjugated which enables detection of immunoblotted target protein bands, without interfering with the immunoprecipitating IgG heavy and light chains has been used. **(D)** Densitometric analysis of immunoprecipitated ITSN in both EC_EH-ITSN_ and EC_Ctrl_ lysates. Data are expressed as ratio of ITSN immunoprecipitated by EHBP1 Ab / ITSN immunoprecipitated by ITSN Ab **(D)**. ^∗^*p* < 0.05. **(E,F)**. Double anti-ITSN Ab anti-rabbit IgG Alexa Fluor 594-conjugated **(E)** / anti-EHBP1 Ab – anti mouse IgG Alexa Fluor 488-conjugated **(F)**. The merged image reveals significant co-localization ITSN/EHBP1, both in the cytosol and at the plasma membrane **(G)**. **(H)** The magnification of the boxed area in **G**, highlights the significant co-localization ITSN/EHBP1 at the plasma membrane level (arrows) and cytosol (arrowheads). Bars: 10 μm **(E–G)**; 5 μm **(H)**; *n* = 5.

As ITSN may bind via its NH_2_-terminal EH domains the EHBP1 to mediate actin reorganization, we examined the potential partnership ITSN/EHBP1 by biochemical and morphological approaches. First, anti-EHBP1 Ab incubated with lysates of EC_Ctrl_ and EC_EH-ITSN_ normalized to 250 μg total protein, brings down not only the EHBP1 (**Figure 6A**) but also ITSN (**Figure 6B**). When lysates of stable transfected EC_EH-ITSN_ were incubated with the anti-EHBP1 Ab, the immunoprecipitates contains the EH_ITSN_ fragment, as well (**Figure 6B**, arrowhead). Moreover, it appears that EHBP1 has a higher affinity for the EH_ITSN_ by comparison to full-length ITSN, as the amount of immunoprecipitated full-length ITSN is slightly lower in EC-EH_ITSN_ compared to EC_Ctrl_. To validate this observation, in parallel experiments we immunoprecipitated ITSN from EC_Ctrl_ and EC-EH_ITSN_ lysates, normalized to 250 μg total protein (**Figure 6C**) using anti-ITSN Ab. The immunoprecipitates were subjected to WB analyses using anti-ITSN Ab followed by densitometric analyses (NIH ImageJ). Accurate assessment of the amounts of ITSN immunoprecipitated by EHBP1 Ab in lysates of EC_Ctrl_ by comparison to EC-EH_ITSN_, was done by expressing the data as ratio of ITSN immunoprecipitated by EHBP1 Ab/ITSN immunoprecipitated by ITSN Ab (**Figure 6D**). The amount of ITSN, immunoprecipitated by EHBP1 from the lysates of EC-EH_ITSN,_ is about 20% lower compared to the amount of ITSN immunoprecipitated by EHBP1 from the lysates of EC_Ctrl._ The finding suggests that the EHBP1 binds preferentially the Myc-EH_ITSN_ by comparison to full-length ITSN.

Double immunofluorescence anti-ITSN-Alexa Fluor 594 / anti-EHBP1-Alexa Fluor 488 reveals for ITSN a strong punctate pattern throughout the cytosol and at the plasma membrane (**Figure 6E**), suggestive of vesicle association and a punctate pattern that overlaps a fine diffuse staining for EHBP1 (**Figure 6F**). Consistent with the immunoprecipitation analysis, a pool of the two proteins co-localize in the cytosol (**Figures 6G,H** arrowheads) as well as in very close proximity of the plasma membrane or at the plasma membrane (**Figures 6G,H** arrows). Significant ITSN/EHBP1 colocalization is detected in the perinuclear area and nucleus (**Figure 6G**).

**FIGURE 7:**
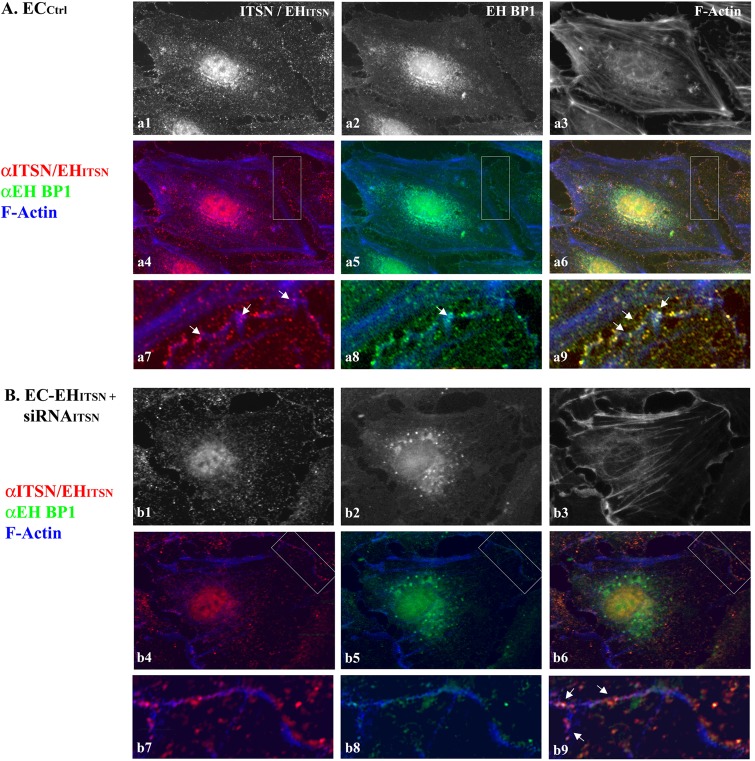
ITSN-EHBP1 interaction contributes to actin organization in ECs. EC_Ctrl_ **(A)** and EC-EH_ITSN_ +siRNA_ITSN_ **(B)** were subjected to tri-color fluorescent staining with anti-ITSN Ab - anti-rabbit IgG Alexa Fluor 594-conjugated **(a1,b1)**, anti-EHBP1 Ab - anti mouse IgG Alexa Fluor 488-conjugated (**a2,b2**) and Phalloidin Alexa-Fluor 350 for detection of F-actin **(a3,b3)**. Merged images anti-ITSN/Phalloidin **(a4,b4)**, anti-EHBP1/Phalloidin **(a5,b5)** and anti-ITSN/anti-EHBP1/Phalloidin **(a6,b6)** were used to analyze the subcellular distribution of ITSN/EHBP1 relative to EC actin cytoskeleton. The magnifications **a7**–**a9** (as indicated by boxed areas) highlight the significant co-localization of ITSN/EHBP1 immunoreactive puncta at the plasma membrane and its proximity. Arrows in **a9** indicate ITSN/EHBP1 immunoreactive puncta very close to actin fibers. When EC-EH_ITSN_ +siRNA_ITSN_ were analyzed, the highly magnified images **b7–b9** (boxed areas in **b4**–**b6**) revealed significantly less ITSN **(b7)** and EHBP1 **(b8)** immunoreactive puncta. The limited ITSN and EHBP1 immunoreactivity is still in very close proximity of actin fibers. Arrows in **b9** indicate ITSN/EHBP1 immunoreactive puncta very close to actin fibers. *n* = 3.

To determine the relationship linking the vesicles containing ITSN with the EHBP1 and cytoskeletal elements we applied three-color staining, anti-ITSN (**Figure 7A-a1**), anti-EHBP1 (**Figure 7A-a2**) and Phalloidin/Alexa Fluor 350 for filamentous (F)-actin detection (**Figure 7A-a3**). In EC_Ctrl_ the endothelial dense peripheral actin band and several stress fibers are easily detected (**Figure 7A-a3**). Both ITSN and EHBP1 immunoreactivities are in close proximity of actin fibers (**Figures 7A-a4–a8**); and limited co-localization with actin is detected, suggesting a possible link of ITSN/EHBP1 complexes to actin fibers, (**Figures 7A-a6,a9**, arrows).

To mimic the disease conditions, we next investigated the distribution of ITSN, EHBP1 and F-actin in stably expressing EH_ITSN_ ECs subjected to ITSN knockdown via the siRNA approach, for 38 h – 40 h as in **Figure 1B**. In these ECs the immunoreactivity for ITSN is considerably lower, **Figure 7B-b1**. The remaining ITSN and the stably expressed Myc-EH_ITSN_ (recognized by the polyclonal ITSN Ab) are randomly distributed as discrete fluorescent puncta in the cytosol and at the plasma membrane, **Figures 7B-b4,b7**) and cytosol. ITSN knocked down altered, however, the distribution of both EHBP1 and actin. Instead of the discrete fluorescent puncta dispersed throughout the cytosol and at the plasma membrane, EHBP1 is found as large fluorescent puncta accumulated in the perinuclear area, **Figures 7B-b2,b5**. However, on the endothelial background, the EHBP1 immunoreactivity at the plasma membrane is very limited, **Figures 7B-b5,b8**. Phalloidin staining revealed the lack of the peripheral dense actin band, the presence of long stress fibers and membrane ruffles (**Figure 7B-b3**). It appears that ITSN expression is required for EHBP1 presence at the plasma membrane. Only several large fluorescent puncta where ITSN colocalizes with the EHBP1 were detected (**Figures 7B-b6,b9** arrows), indicating a possible association of ITSN/EHBP1 complexes to actin fibers. Interestingly, despite ITSN deficiency, ECs expressing the EH_ITSN_ (**Figures 7B-b1,b4**) still display nuclear immunoreactivity to ITSN Ab, most likely, the result of nuclear localization of the exogenously expressed EH_ITSN._ The observation is very suggestive of potential functional implications of EH_ITSN_ localization in the nucleus.

These observations suggest that ITSN and EHBP1 are components of a complex with essential functions in both the organization of cell membrane associated cytoskeleton and endocytosis. It appears that ITSN recruits the EHBP1 to the plasma membrane where ITSN/EHBP1 complexes can associate with actin and play a role in actin polymerization and formation of dense peripheral actin band. Moreover, lack of the peripheral actin band detected in the ITSN-deficient ECs stably expressing the EH_ITSN_ may favor the increased occurrence of alternative endocytic structures and their intracellular trafficking.

## Discussion

Hyperproliferation, apoptosis-resistance, dysfunctional endocytosis, altered intracellular protein trafficking and increased permeability are characteristics of pulmonary ECs in PAH (Sehgal and Lee, 2011; Long et al., 2015; Zhou et al., 2016). Several studies implicated caveolae and caveolin-1 in EC dysfunction and the pathogenesis of PAH. Mice deficient of caveolin-1 spontaneously develop PAH (Zhao et al., 2002, 2009). Heterozygous mutations in the caveolin-1 gene with distinct effects on caveolae assembly and caveolin-1 trafficking have been identified in patients with PAH (Copeland et al., 2017). It has also been reported that heterozygous null BMPR2 mutations promote caveolae trafficking defects (Prewitt et al., 2015).

Our studies also indicated that the lung tissue of human and animal models of PAH is deficient of ITSN and expresses a biologically active ITSN fragment which triggers the endothelial proliferative phenotype responsible for the severe plexiform arteriopathy (Patel et al., 2013). Deficiency of ITSN, a general endocytic scaffold and regulator of the membrane fission GTPase, dynamin, decreases significantly caveolae number (Knezevic et al., 2011; Predescu et al., 2012). Compelling evidence demonstrates that transendothelial transport/ transcytosis mediated by caveolae is a primary determinant of the basal lung endothelial permeability properties (Sun et al., 2011). Disruption of caveolae function alters the endothelial barrier integrity, enhances vascular permeability and upregulates compensatory endocytic pathways and their morphological intermediates, usually underrepresented (Drab et al., 2001; Miyawaki-Shimizu et al., 2006; Predescu et al., 2012, 2013). Our present studies extend these observations as we found that prolonged EH_ITSN_ expression cooperates with ITSN deficiency in EC_PAH_ and experimental animal models of PAH in decreasing caveolae number, impairing their transport function while boosting the upregulation of alternative transport pathways. Thus, due to these features, the experiments on EC_PAH_ cells, best reflect the human disease condition and also the 2-hit mouse model of plexiform arteriopathy we generated. We demonstrate using EC_KD-ITSN_ and EC_PAH_ that the non-conventional structures are involved in biotin internalization and can compensate for deficient endocytosis via caveolae. Pulmonary ECs of EH_ITSN_-transduced K0^*ITSN+/-*^ mice show decreased number of caveolae and increased occurrence of caveolae clusters, tubulo-vesicular and enlarged endocytic structures and membranous rings. Moreover, using gold tagged-BSA perfusion of the mouse and rat lung microvasculature, we demonstrate that these non-conventional structures are involved in the uptake, internalization and transendothelial transport. Our data prove that the upregulated alternative pathways may functionally rescue the transport and nutrient uptake, usually mediated by the endothelial caveolae. This observation demonstrate that EH_ITSN_ expression, a 2nd component or 2nd “hit” mechanism generated in response to pathological risk factors (inflammation, increase CD8 + lymphocyte, granzyme B secretion), with no drug administration or hypoxia exposure, is required to drive not only formation of plexiform lesions but also to “bounce” the endocytic activity of dysfunctional ECs in order to support their hyper-proliferation and metabolic activity.

However, the lack of caveolae and as results the lack of the canonical caveolar signaling pathways responsible for controlling the understudied and less scrutinized molecular mechanisms governing the cross-talk between the caveolae and the non-conventional upregulated structures have detrimental consequences on EC function. This emerging phenotype characterized by loss of caveolae in favor of alternative pathways, cannot, however, substitute for endocytosis involvement in cellular proliferation (Sigismund et al., 2012), and therefore the new feature – EC hyper proliferation – is first to be noticed. We have also shown that in pathological conditions, dysfunctional ECs show altered intracellular trafficking and signaling of cell surface receptors such as the TGFβR1 (Bardita et al., 2015), implicated in the pathogenesis of PAH (Garcia-Rivas et al., 2017; Graf et al., 2018). Compelling evidence indicates the essential involvement of different endocytic routes (caveolae, clathrin-mediated, lipid rafts) in cellular signaling events with consequences on disease-linked phenotypes. Heterozygous germline mutations in BMPRII, such as those observed in patients with familial PAH, contribute to the failure of the receptor to traffic routinely from the ER to Golgi and reach the cell surface; the intracellular withholding, lack of access to the extracellular ligand and endocytic internalization led to altered receptor signaling in response to BMPs (Sobolewski et al., 2008; Morrell, 2010). Multiple dysfunctions in the endocytic (anterograde and retrograde) vesicular trafficking, abnormal trafficking of relevant receptors (i.e., BMPRI, BMPRII, IL-6R, gp130) and vasoactive mediators via alternative pathways that bypass the Golgi apparatus, have been implicated in the pathogenesis of PAH, as well (Sehgal and Lee, 2011). Thus, the increasing occurrence of the alternative endocytic structures may alter the subcellular localization of cell surface receptors, their signaling, their fate and sorting with functional consequences for proteins involved (Di Fiore and De Camilli, 2001; Le Roy and Wrana, 2005; Bardita et al., 2015).

Our studies also demonstrate that ITSN-deficiency and the EH_ITSN_ presence promote actin reorganization leading to the widening and leakiness of the intercellular junctions. The resulting loss of endothelial barrier integrity provides increased sensitivity for proliferative and inflammatory mediators to reach the medial and adventitial vascular layers, leading to cell proliferation and PAH progression. The studies reporting a link between a dysfunctional lung endothelial barrier and PAH pathogenesis are limited. Lung microvascular leak has been associated with lung cell hyperplasia and right ventricular hypertrophy in both MCT- and hypoxia-induced PAH (Sugita et al., 1983). More recently, Zhou et al. (2016) reported that animals with severe PAH display increased sensitivity to vascular permeability induced by activation of store-operated calcium entry. Additional studies demonstrated a role for endothelial BMPRII loss in causing increased vascular permeability and altered translocation across the vascular wall (Burton et al., 2011a,b; Kim et al., 2013). Our data are evidence that ECs, as the critical initiating cell type in PAH, can contribute to the PAH pathology not only by increased apoptosis followed by exuberant proliferation, but also by increased vascular permeability.

Actin reorganization, the formation of stress fibers and lack of cortical actin disturb not only the proper interaction of ECs with each other and their interaction with the micro-environment but also the normal endocytic membrane trafficking. We found that ITSN-deficiency and expression of the EH_ITSN_ alter the subcellular distribution of the EHBP1, a protein that functions near the plasma membrane and couples endocytosis to the actin cytoskeleton (Guilherme et al., 2004). EHBP1 is expressed in EC_S_ and interacts with ITSN via the NH_2_-terminal EH domains. Within full-length ITSN, the EH_ITSN_ are under the inhibitory control of the SH3A–E domains that may account for the spatial and temporal coordination of cellular activities in specific location within the cell (Adams et al., 2000). The free EH_ITSN_ escapes the regulatory control of the SH3A–E, and as result, its interaction with the EHBP1 is augmented. ITSN deficiency and thus, the lack of its scaffolding properties, prevent the proper EHBP1 localization at its site of action. As a result, the organization of cortical actin cytoskeleton is impaired favoring the formation and motility of tubulo-vesicular and enlarged endocytic structures and membranous rings, and thus allow to compensate for deficient caveolae intracellular transport. We have previously shown that actin polymerization and remodeling sub-adjacent to the plasma membrane decrease caveolae endocytosis significantly in ECs (Klein et al., 2009). We have also shown that ITSN deficiency is a crucial player in Rac1 activation and vimentin filament organization (Jeganathan et al., 2016). This is significant as Rac1 and vimentin are essential regulators of the cytoskeleton structure with control over the epithelial-mesenchymal transition, cell migration and metastasis of lung cancer (Jeganathan et al., 2016). The observations raised the possibility that ITSN deficiency and EH_ITSN_ expression resulting in an EC proliferative phenotype and loss of junctional integrity may provoke ECs to acquire a mesenchymal phenotype. The mesenchymal EC phenotype is present in experimental PAH animal models and has significant contribution to tissue regeneration and vascular remodeling in PAH (Good et al., 2015; Ranchoux et al., 2015; Tang et al., 2018).

## Conclusion

We show that the EH_ITSN_ besides contributing to the proliferation and overgrowth of ECs, triggers the upregulation of the morpho-functional alternative endocytic activity of dysfunctional EC_PAH_, and hence, play a role in the development and maintenance EC_PAH_ phenotype.

## Author Contributions

DP and SP conceived and designed the research, analyzed the data, interpreted the results of experiments, and drafted the manuscript. DP, MP, SQ, CB, and RB performed the experiments. MP, SQ, and DP prepared the figures. SP edited and revised the manuscript.

## Conflict of Interest Statement

The authors declare that the research was conducted in the absence of any commercial or financial relationships that could be construed as a potential conflict of interest.

## Footnotes

1 http://www.psi.ch/lbr/SquasshAnalystEN/SquasshAnalyst.zip

2 http://fiji.sc/

## References

[B1] Adams A. Thorn J. M. Yamabhai M. Kay B. K. O’Bryan J. P. (2000). Intersectin, an adaptor protein involved in clathrin-mediated endocytosis, activates mitogenic signaling pathways. *J. Biol. Chem.* 275 27414–27420. 10.1074/jbc.M004810200 10851244

[B2] Bardita C. Predescu D. N. Sha F. Patel M. Balaji G. Predescu S. A. (2015). Endocytic deficiency induced by ITSN-1s knockdown alters the Smad2/3-Erk1/2 signaling balance downstream of Alk5. *J. Cell Sci.* 128 1528–1541. 10.1242/jcs.163030 25720380 PMC4406123

[B3] Burton V. J. Ciuclan L. I. Holmes A. M. Rodman D. M. Walker C. Budd D. C. (2011a). Bone morphogenetic protein receptor II regulates pulmonary artery endothelial cell barrier function. *Blood* 117 333–341. 10.1182/blood-2010-05-285973 20724539

[B4] Burton V. J. Holmes A. M. Ciuclan L. I. Robinson A. Roger J. S. Jarai G. (2011b). Attenuation of leukocyte recruitment via CXCR1/2 inhibition stops the progression of PAH in mice with genetic ablation of endothelial BMPR-II. *Blood* 118 4750–4758. 10.1182/blood-2011-05-347393 21900197 PMC3208288

[B5] Chazova I. Loyd J. E. Zhdanov V. S. Newman J. H. Belenkov Y. Meyrick B. (1995). Pulmonary artery adventitial changes and venous involvement in primary pulmonary hypertension. *Am. J. Pathol.* 146 389–397. 7856750 PMC1869854

[B6] Comhair S. A. Xu W. Mavrakis L. Aldred M. A. Asosingh K. Erzurum S. C. (2012). Human primary lung endothelial cells in culture. *Am. J. Respir. Cell Mol. Biol.* 46 723–730. 10.1165/rcmb.2011-0416TE 22427538 PMC3380284

[B7] Copeland C. A. Han B. Tiwari A. Austin E. D. Loyd J. E. West J. D. (2017). A disease-associated frameshift mutation in caveolin-1 disrupts caveolae formation and function through introduction of a de novo ER retention signal. *Mol. Biol. Cell* 28 3095–3111. 10.1091/mbc.E17-06-0421 28904206 PMC5662265

[B8] de Beer T. Carter R. E. Lobel-Rice K. E. Sorkin A. Overduin M. (1998). Structure and Asn-Pro-Phe binding pocket of the Eps15 homology domain. *Science* 281 1357–1360. 10.1126/science.281.5381.1357 9721102

[B9] Di Fiore P. P. De Camilli P. (2001). Endocytosis and signaling. an inseparable partnership. *Cell* 106 1–4. 10.1016/S0092-8674(01)00428-7 11461694

[B10] Drab M. Verkade P. Elger M. Kasper M. Lohn M. Lauterbach B. (2001). Loss of caveolae, vascular dysfunction, and pulmonary defects in caveolin-1 gene-disrupted mice. *Science* 293 2449–2452. 10.1126/science.1062688 11498544

[B11] Fernandez-Chacon R. Achiriloaie M. Janz R. Albanesi J. P. Sudhof T. C. (2000). SCAMP1 function in endocytosis. *J. Biol. Chem.* 275 12752–12756. 10.1074/jbc.275.17.12752 10777571

[B12] Garcia-Rivas G. Jerjes-Sanchez C. Rodriguez D. Garcia-Pelaez J. Trevino V. (2017). A systematic review of genetic mutations in pulmonary arterial hypertension. *BMC Med. Genet.* 18:82. 10.1186/s12881-017-0440-5 28768485 PMC5541665

[B13] Good R. B. Gilbane A. J. Trinder S. L. Denton C. P. Coghlan G. Abraham D. J. (2015). Endothelial to mesenchymal transition contributes to endothelial dysfunction in pulmonary arterial hypertension. *Am. J. Pathol.* 185 1850–1858. 10.1016/j.ajpath.2015.03.019 25956031

[B14] Graf S. Haimel M. Bleda M. Hadinnapola C. Southgate L. Li W. (2018). Identification of rare sequence variation underlying heritable pulmonary arterial hypertension. *Nat. Commun.* 9:1416. 10.1038/s41467-018-03672-4 29650961 PMC5897357

[B15] Guilherme A. Soriano N. A. Bose S. Holik J. Bose A. Pomerleau D. P. (2004). EHD2 and the novel EH domain binding protein EHBP1 couple endocytosis to the actin cytoskeleton. *J. Biol. Chem.* 279 10593–10605. 10.1074/jbc.M307702200 14676205

[B16] Jeganathan N. Predescu D. Zhang J. Sha F. Bardita C. Patel M. (2016). Rac1-mediated cytoskeleton rearrangements induced by intersectin-1s deficiency promotes lung cancer cell proliferation, migration and metastasis. *Mol. Cancer* 15:59. 10.1186/s12943-016-0543-1 27629044 PMC5024437

[B17] Johnson J. A. Hemnes A. R. Perrien D. S. Schuster M. Robinson L. J. Gladson S. (2012). Cytoskeletal defects in Bmpr2-associated pulmonary arterial hypertension. *Am. J. Physiol. Lung Cell Mol. Physiol.* 302 L474–L484. 10.1152/ajplung.00202.2011 22180660 PMC3311512

[B18] Keren T. Roth M. G. Henis Y. I. (2001). Internalization-competent influenza hemagglutinin mutants form complexes with clathrin-deficient multivalent AP-2 oligomers in live cells. *J. Biol. Chem.* 276 28356–28363. 10.1074/jbc.M102235200 11369772

[B19] Kim C. W. Song H. Kumar S. Nam D. Kwon H. S. Chang K. H. (2013). Anti-inflammatory and antiatherogenic role of BMP receptor II in endothelial cells. *Arterioscler. Thromb. Vasc. Biol.* 33 1350–1359. 10.1161/ATVBAHA.112.300287 23559633 PMC3758923

[B20] Klein I. K. Predescu D. N. Sharma T. Knezevic I. Malik A. B. Predescu S. (2009). Intersectin-2L regulates caveola endocytosis secondary to Cdc42-mediated actin polymerization. *J. Biol. Chem.* 284 25953–25961. 10.1074/jbc.M109.035071 19622753 PMC2757996

[B21] Knezevic I. Predescu D. Bardita C. Wang M. Sharma T. Keith B. (2011). Regulation of dynamin-2 assembly-disassembly and function through the SH3A domain of intersectin-1s. *J. Cell Mol. Med.* 15 2364–2376. 10.1111/j.1582-4934.2010.01226.x 21129155 PMC3072443

[B22] Lachmanovich E. Shvartsman D. E. Malka Y. Botvin C. Henis Y. I. Weiss A. M. (2003). Co-localization analysis of complex formation among membrane proteins by computerized fluorescence microscopy: application to immunofluorescence co-patching studies. *J. Microsc.* 212 122–131. 10.1046/j.1365-2818.2003.01239.x 14629561

[B23] Le Roy C. Wrana J. L. (2005). Clathrin- and non-clathrin-mediated endocytic regulation of cell signalling. *Nat. Rev. Mol. Cell Biol.* 6 112–126. 10.1038/nrm1571 15687999

[B24] Loeb C. R. Harris J. L. Craik C. S. (2006). Granzyme B proteolyzes receptors important to proliferation and survival, tipping the balance towards apoptosis. *J. Biol. Chem.* 281 28326–28335. 10.1074/jbc.M604544200 16798735

[B25] Long L. Ormiston M. L. Yang X. Southwood M. Graf S. Machado R. D. (2015). Selective enhancement of endothelial BMPR-II with BMP9 reverses pulmonary arterial hypertension. *Nat. Med.* 21 777–785. 10.1038/nm.3877 26076038 PMC4496295

[B26] Martina J. A. Bonangelino C. J. Aguilar R. C. Bonifacino J. S. (2001). Stonin 2: an adaptor-like protein that interacts with components of the endocytic machinery. *J. Cell Biol.* 153 1111–1120. 10.1083/jcb.153.5.1111 11381094 PMC2174325

[B27] Miyawaki-Shimizu K. Predescu D. Shimizu J. Broman M. Predescu S. Malik A. B. (2006). siRNA-induced caveolin-1 knockdown in mice increases lung vascular permeability via the junctional pathway. *Am. J. Physiol. Lung Cell Mol. Physiol.* 290 L405–L413. 10.1152/ajplung.00292.2005 16183667

[B28] Morrell N. W. (2010). Role of bone morphogenetic protein receptors in the development of pulmonary arterial hypertension. *Adv. Exp. Med. Biol.* 661 251–264. 10.1007/978-1-60761-500-2_16 20204735

[B29] Mukherjee S. Tessema M. Wandinger-Ness A. (2006). Vesicular trafficking of tyrosine kinase receptors and associated proteins in the regulation of signaling and vascular function. *Circ. Res.* 98 743–756. 10.1161/01.RES.0000214545.99387.e3 16574915

[B30] Palade G. E. Bruns R. R. (1968). Structural modulations of plasmalemmal vesicles. *J. Cell Biol.* 37 633–649. 10.1083/jcb.37.3.633 11905197 PMC2107438

[B31] Patel M. Predescu D. Bardita C. Chen J. Jeganathan N. Pritchard M. (2017). Modulation of intersectin-1s lung expression induces obliterative remodeling and severe plexiform arteriopathy in the murine pulmonary vascular bed. *Am. J. Pathol.* 187 528–542. 10.1016/j.ajpath.2016.11.012 28068512 PMC5389368

[B32] Patel M. Predescu D. N. Tandon R. Bardita C. Pogoriler J. Bhorade S. (2013). A novel p38 mitogen-activated protein kinase/Elk-1-dependent molecular mechanism underlying abnormal endothelial cell proliferation in plexogenic pulmonary arterial hypertension. *J. Biol. Chem.* 288 25701–25716. 10.1074/jbc.M113.502674 23893408 PMC3764778

[B33] Polo S. Confalonieri S. Salcini A. E. Di Fiore P. P. (2003). EH and UIM: endocytosis and more. *Sci. STKE* 2003:re17. 10.1126/stke.2132003re17 14679291

[B34] Polo S. Di Fiore P. P. (2006). Endocytosis conducts the cell signaling orchestra. *Cell* 124 897–900. 10.1016/j.cell.2006.02.025 16530038

[B35] Predescu D. Horvat R. Predescu S. Palade G. E. (1994). Transcytosis in the continuous endothelium of the myocardial microvasculature is inhibited by N-ethylmaleimide. *Proc. Natl. Acad. Sci. U.S.A.* 91 3014–3018. 10.1073/pnas.91.8.3014 8159697 PMC43505

[B36] Predescu D. Predescu S. McQuistan T. Palade G. E. (1998). Transcytosis of alpha1-acidic glycoprotein in the continuous microvascular endothelium. *Proc. Natl. Acad. Sci. U.S.A.* 95 6175–6180. 10.1073/pnas.95.11.6175 9600937 PMC27616

[B37] Predescu D. N. Bardita C. Tandon R. Predescu S. A. (2013). Intersectin-1s: an important regulator of cellular and molecular pathways in lung injury. *Pulm. Circ.* 3 478–498. 10.1086/674439 24618535 PMC4070809

[B38] Predescu D. N. Neamu R. Bardita C. Wang M. Predescu S. A. (2012). Impaired caveolae function and upregulation of alternative endocytic pathways induced by experimental modulation of intersectin-1s expression in mouse lung endothelium. *Biochem. Res. Int.* 2012:672705. 10.1155/2012/672705 22506115 PMC3299393

[B39] Predescu S. A. Predescu D. N. Knezevic I. Klein I. K. Malik A. B. (2007a). Intersectin-1s regulates the mitochondrial apoptotic pathway in endothelial cells. *J. Biol. Chem.* 282 17166–17178. 17405881 10.1074/jbc.M608996200

[B40] Predescu S. A. Predescu D. N. Malik A. B. (2007b). Molecular determinants of endothelial transcytosis and their role in endothelial permeability. *Am. J. Physiol. Lung Cell Mol. Physiol.* 293 L823–L842. 10.1152/ajplung.00436.2006 17644753

[B41] Predescu S. A. Predescu D. N. Palade G. E. (1997). Plasmalemmal vesicles function as transcytotic carriers for small proteins in the continuous endothelium. *Am. J. Physiol.* 272(2 Pt 2), H937–H949. 9124458 10.1152/ajpheart.1997.272.2.H937

[B42] Predescu S. A. Predescu D. N. Palade G. E. (2001). Endothelial transcytotic machinery involves supramolecular protein-lipid complexes. *Mol. Biol. Cell* 12 1019–1033. 10.1091/mbc.12.4.1019 11294904 PMC32284

[B43] Predescu S. A. Predescu D. N. Timblin B. K. Stan R. V. Malik A. B. (2003). Intersectin regulates fission and internalization of caveolae in endothelial cells. *Mol. Biol. Cell* 14 4997–5010. 10.1091/mbc.e03-01-0041 12960435 PMC284801

[B44] Prewitt A. R. Ghose S. Frump A. L. Datta A. Austin E. D. Kenworthy A. K. (2015). Heterozygous null bone morphogenetic protein receptor type 2 mutations promote SRC kinase-dependent caveolar trafficking defects and endothelial dysfunction in pulmonary arterial hypertension. *J. Biol. Chem.* 290 960–971. 10.1074/jbc.M114.591057 25411245 PMC4294523

[B45] Ranchoux B. Antigny F. Rucker-Martin C. Hautefort A. Pechoux C. Bogaard H. J. (2015). Endothelial-to-mesenchymal transition in pulmonary hypertension. *Circulation* 131 1006–1018. 10.1161/CIRCULATIONAHA.114.008750 25593290

[B46] Rothberg K. G. Heuser J. E. Donzell W. C. Ying Y. S. Glenney J. R. Anderson R. G. (1992). Caveolin, a protein component of caveolae membrane coats. *Cell* 68 673–682. 10.1016/0092-8674(92)90143-Z 1739974

[B47] Sakao S. Taraseviciene-Stewart L. Lee J. D. Wood K. Cool C. D. Voelkel N. F. (2005). Initial apoptosis is followed by increased proliferation of apoptosis-resistant endothelial cells. *FASEB J.* 19 1178–1180. 10.1096/fj.04-3261fje 15897232

[B48] Sehgal P. B. Lee J. E. (2011). Protein trafficking dysfunctions: role in the pathogenesis of pulmonary arterial hypertension. *Pulm. Circ.* 1 17–32. 10.4103/2045-8932.78097 22034594 PMC3198637

[B49] Sigismund S. Confalonieri S. Ciliberto A. Polo S. Scita G. Di Fiore P. P. (2012). Endocytosis and signaling: cell logistics shape the eukaryotic cell plan. *Physiol. Rev.* 92 273–366. 10.1152/physrev.00005.2011 22298658 PMC5614474

[B50] Simionescu M. Gafencu A. Antohe F. (2002). Transcytosis of plasma macromolecules in endothelial cells: a cell biological survey. *Microsc. Res. Tech.* 57 269–288. 10.1002/jemt.10086 12112439

[B51] Sobolewski A. Rudarakanchana N. Upton P. D. Yang J. Crilley T. K. Trembath R. C. (2008). Failure of bone morphogenetic protein receptor trafficking in pulmonary arterial hypertension: potential for rescue. *Hum. Mol. Genet.* 17 3180–3190. 10.1093/hmg/ddn214 18647753

[B52] Sorkin A. von Zastrow M. (2009). Endocytosis and signalling: intertwining molecular networks. *Nat. Rev. Mol. Cell Biol.* 10 609–622. 10.1038/nrm2748 19696798 PMC2895425

[B53] Stenmark K. R. Meyrick B. Galie N. Mooi W. J. McMurtry I. F. (2009). Animal models of pulmonary arterial hypertension: the hope for etiological discovery and pharmacological cure. *Am. J. Physiol. Lung Cell Mol. Physiol.* 297 L1013–L1032. 10.1152/ajplung.00217.2009 19748998

[B54] Stevens T. Gillespie M. N. (2007). The hyperproliferative endothelial cell phenotype in idiopathic pulmonary arterial hypertension. *Am. J. Physiol. Lung Cell Mol. Physiol.* 293 L546–L547. 10.1152/ajplung.00246.2007 17601794

[B55] Stoeber M. Stoeck I. K. Hanni C. Bleck C. K. Balistreri G. Helenius A. (2012). Oligomers of the ATPase EHD2 confine caveolae to the plasma membrane through association with actin. *EMBO J.* 31 2350–2364. 10.1038/emboj.2012.98 22505029 PMC3364743

[B56] Sugita T. Hyers T. M. Dauber I. M. Wagner W. W. McMurtry I. F. Reeves J. T. (1983). Lung vessel leak precedes right ventricular hypertrophy in monocrotaline-treated rats. *J. Appl. Physiol. Respir. Environ. Exerc. Physiol.* 54 371–374. 10.1152/jappl.1983.54.2.371 6219974

[B57] Sun Y. Minshall R. D. Hu G. (2011). Role of caveolin-1 in the regulation of pulmonary endothelial permeability. *Methods Mol. Biol.* 763 303–317. 10.1007/978-1-61779-191-8_21 21874461

[B58] Tang H. Babicheva A. McDermott K. M. Gu Y. Ayon R. J. Song S. (2018). Endothelial HIF-2alpha contributes to severe pulmonary hypertension due to endothelial-to-mesenchymal transition. *Am. J. Physiol. Lung Cell Mol. Physiol.* 314 L256–L275. 10.1152/ajplung.00096.2017 29074488 PMC5866501

[B59] Tuder R. M. Abman S. H. Braun T. Capron F. Stevens T. Thistlethwaite P. A. (2009). Development and pathology of pulmonary hypertension. *J. Am. Coll. Cardiol.* 54(Suppl.), S3–S9. 10.1016/j.jacc.2009.04.009 19555856

[B60] Tuder R. M. Cool C. D. Yeager M. Taraseviciene-Stewart L. Bull T. M. Voelkel N. F. (2001). The pathobiology of pulmonary hypertension. Endothelium. *Clin. Chest Med.* 22 405–418. 10.1016/S0272-5231(05)70280-X 11590837

[B61] Tuder R. M. Groves B. Badesch D. B. Voelkel N. F. (1994). Exuberant endothelial cell growth and elements of inflammation are present in plexiform lesions of pulmonary hypertension. *Am. J. Pathol.* 144 275–285. 7508683 PMC1887146

[B62] Tuder R. M. Marecki J. C. Richter A. Fijalkowska I. Flores S. (2007). Pathology of pulmonary hypertension. *Clin. Chest Med.* 28:23–vii. 10.1016/j.ccm.2006.11.010 17338926 PMC1924722

[B63] Vieira A. V. Lamaze C. Schmid S. L. (1996). Control of EGF receptor signaling by clathrin-mediated endocytosis. *Science* 274 2086–2089. 10.1126/science.274.5295.2086 8953040

[B64] Yamabhai M. Hoffman N. G. Hardison N. L. McPherson P. S. Castagnoli L. Cesareni G. (1998). Intersectin, a novel adaptor protein with two Eps15 homology and five Src homology 3 domains. *J. Biol. Chem.* 273 31401–31407. 10.1074/jbc.273.47.31401 9813051

[B65] Yap C. C. Lasiecka Z. M. Caplan S. Winckler B. (2010). Alterations of EHD1/EHD4 protein levels interfere with L1/NgCAM endocytosis in neurons and disrupt axonal targeting. *J. Neurosci.* 30 6646–6657. 10.1523/JNEUROSCI.5428-09.2010 20463227 PMC2905050

[B66] Zhao Y. Y. Liu Y. Stan R. V. Fan L. Gu Y. Dalton N. (2002). Defects in caveolin-1 cause dilated cardiomyopathy and pulmonary hypertension in knockout mice. *Proc. Natl. Acad. Sci. U.S.A.* 99 11375–11380. 10.1073/pnas.172360799 12177436 PMC123264

[B67] Zhao Y. Y. Zhao Y. D. Mirza M. K. Huang J. H. Potula H. H. Vogel S. M. (2009). Persistent eNOS activation secondary to caveolin-1 deficiency induces pulmonary hypertension in mice and humans through PKG nitration. *J. Clin. Invest.* 119 2009–2018. 10.1172/JCI33338 19487814 PMC2701851

[B68] Zhou C. Townsley M. I. Alexeyev M. Voelkel N. F. Stevens T. (2016). Endothelial hyperpermeability in severe pulmonary arterial hypertension: role of store-operated calcium entry. *Am. J. Physiol. Lung Cell Mol. Physiol.* 311 L560–L569. 10.1152/ajplung.00057.2016 27422996 PMC5142214

