# Supplementary material for: Epsin15 Homology Domains: Role in the Pathogenesis of Pulmonary Arterial Hypertension
Source: Front Physiol. 2018 Oct 2;9:1393. doi: 10.3389/fphys.2018.01393 (PMC6176378; doi:10.3389/fphys.2018.01393)

**Epsin15 homology domains: role in the pathogenesis of pulmonary arterial hypertension**

Dan Predescu^§^, Shanshan Qin^§^, Monal Patel^¥^, Cristina Bardita^£^, Rabia Bhalli and Sanda Predescu*

Rush University Medical College, Department of Internal Medicine, Division of Pulmonary, Critical Care and Sleep Medicine, Chicago, IL

[Dan_Predescu@rush.edu](mailto:Dan_Predescu@rush.edu); [Shanshan_Qin@rush.edu](mailto:Shanshan_Qin@rush.edu); [mons2k@yahoo.com](mailto:mons2k@yahoo.com); [bardita@uabmc.edu](mailto:cbardita@uabmc.edu); [Rabia.Zahid@hotmail.com](mailto:Rabia.Zahid@hotmail.com); [Sanda_Predescu@rush.edu](mailto:Sanda_Predescu@rush.edu)

***Corresponding Author:**

Sanda Predescu, PhD, FAHA

[Sanda_Predescu@rush.edu](mailto:Sanda_Predescu@rush.edu)

^§^ DP and SQ contributed equally to this work.

Present address: ^¥^Division of Hematology/Oncology; Robert H. Lurie Comprehensive Cancer Center, Northwestern University Feinberg School of Medicine; ^£^Pulmonary, Allergy and Critical Care Medicine University of Alabama at Birmingham, Birmingham, AL.

**Supplementary Figure 1**

1. K0*^ITSN+/-^* mice were selected by genotyping; conventional RT-PCR applied on K0*^ITSN+/-^* mouse lung samples, followed by densitometry. Cyclophilin was used as an internal control. WB analyses of ITSN protein expression in the K0*^ITSN+/-^* mice compared to wt-mice followed by densitometric analyses. n=9.
2. Efficient expression of the EH_ITSN_ for 21 days was achieved by repeated delivery of the myc-EH_ITSN_. Actin was used as a loading control; n=3.

**C, D.** Right ventricular systolic pressure (RVSP) and Fulton’s index: [RV / (LV + S)] were determined as in ([Patel et al., 2017](#_ENREF_31)); n=9.

**E, F.** The lung tissue of both MCT-mice and rats is deficient of ITSN and shows expression of the EH_ITSN_; Actin was used as a loading control; n=3. *p <0.05; **p<0.01.


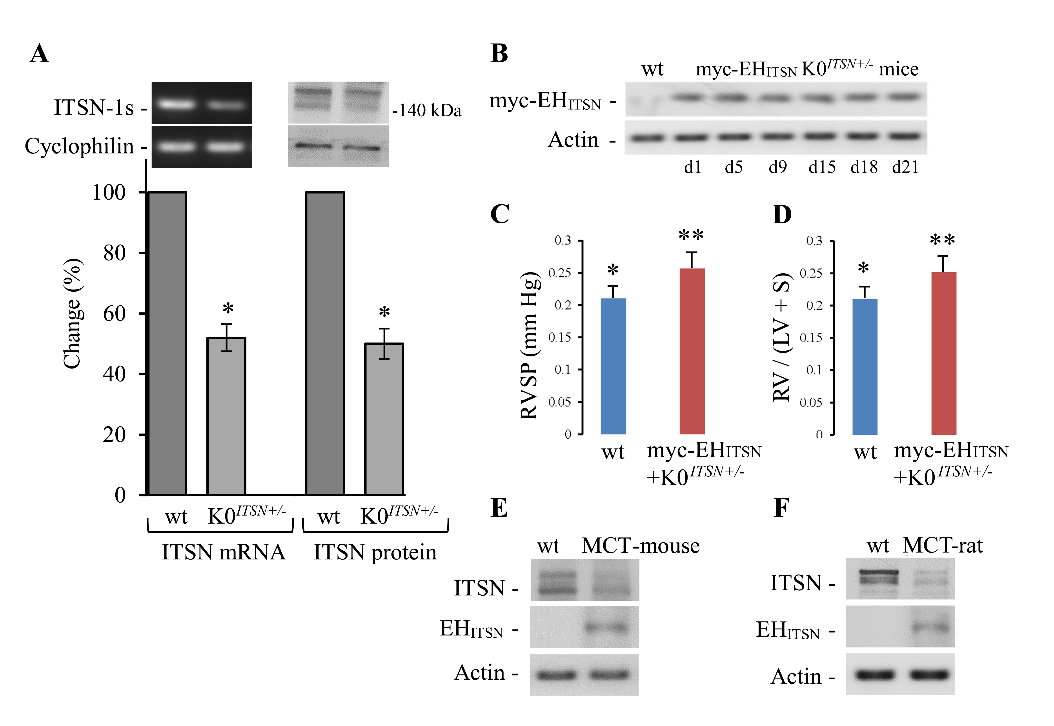

Supplement: Supplementary file 1 [file Data_Sheet_1.docx]
